# Supplementary material for: Multi‐dataset identification of innovative feature genes and molecular mechanisms in keratoconus
Source: J Cell Mol Med. 2024 Sep 19;28(18):e70079. doi: 10.1111/jcmm.70079 (PMC11412914; doi:10.1111/jcmm.70079)
Supplement: Supplementary file 2 — Material S2. [file JCMM-28-e70079-s002.zip › 7.GSEA analysis/4.MYRF/KEGG_MATURITY_ONSET_DIABETES_OF_THE_YOUNG.html]

Details for gene set KEGG\_MATURITY\_ONSET\_DIABETES\_OF\_THE\_YOUNG[GSEA]

|  || Dataset | MYRF.group.cls#h\_versus\_l |
| Phenotype | group.cls#h\_versus\_l |
| Upregulated in class | h |
| GeneSet | KEGG\_MATURITY\_ONSET\_DIABETES\_OF\_THE\_YOUNG |
| Enrichment Score (ES) | 0.23467706 |
| Normalized Enrichment Score (NES) | 0.6156578 |
| Nominal p-value | 0.99 |
| FDR q-value | 1.0 |
| FWER p-Value | 1.0 |
Table: GSEA Results Summary

  

Fig 1: Enrichment plot: KEGG\_MATURITY\_ONSET\_DIABETES\_OF\_THE\_YOUNG      
 Profile of the Running ES Score & Positions of GeneSet Members on the Rank Ordered List

  

| SYMBOL | TITLE | RANK IN GENE LIST | RANK METRIC SCORE | RUNNING ES | CORE ENRICHMENT || 1 | NKX6-1 | na | 996 | 0.461 | 0.0868 | Yes |
| 2 | HNF1A | na | 2744 | 0.309 | 0.1140 | Yes |
| 3 | BHLHA15 | na | 3577 | 0.268 | 0.1572 | Yes |
| 4 | HHEX | na | 3891 | 0.258 | 0.2128 | Yes |
| 5 | SLC2A2 | na | 5961 | 0.194 | 0.2017 | Yes |
| 6 | NKX2-2 | na | 7589 | 0.149 | 0.1920 | Yes |
| 7 | PAX6 | na | 8335 | 0.131 | 0.2033 | Yes |
| 8 | PDX1 | na | 8376 | 0.130 | 0.2347 | Yes |
| 9 | HNF1B | na | 10542 | 0.086 | 0.1938 | No |
| 10 | IAPP | na | 11212 | 0.075 | 0.1933 | No |
| 11 | FOXA3 | na | 12106 | 0.062 | 0.1831 | No |
| 12 | PAX4 | na | 12539 | 0.056 | 0.1847 | No |
| 13 | FOXA2 | na | 15810 | 0.020 | 0.0954 | No |
| 14 | NEUROG3 | na | 16626 | 0.012 | 0.0748 | No |
| 15 | MAFA | na | 18201 | -0.003 | 0.0301 | No |
| 16 | NEUROD1 | na | 20262 | -0.025 | -0.0230 | No |
| 17 | NR5A2 | na | 23591 | -0.069 | -0.1016 | No |
| 18 | MNX1 | na | 25138 | -0.096 | -0.1222 | No |
| 19 | PKLR | na | 27534 | -0.149 | -0.1539 | No |
| 20 | HES1 | na | 28151 | -0.165 | -0.1304 | No |
| 21 | HNF4G | na | 29062 | -0.193 | -0.1083 | No |
| 22 | INS | na | 30231 | -0.232 | -0.0839 | No |
| 23 | HNF4A | na | 30314 | -0.234 | -0.0275 | No |
| 24 | ONECUT1 | na | 31576 | -0.294 | 0.0098 | No |
| 25 | GCK | na | 32098 | -0.323 | 0.0758 | No |
Table: GSEA details [plain text format]

  

Fig 2: KEGG\_MATURITY\_ONSET\_DIABETES\_OF\_THE\_YOUNG      
 Blue-Pink O' Gram in the Space of the Analyzed GeneSet

  

Fig 3: KEGG\_MATURITY\_ONSET\_DIABETES\_OF\_THE\_YOUNG: Random ES distribution      
 Gene set null distribution of ES for **KEGG\_MATURITY\_ONSET\_DIABETES\_OF\_THE\_YOUNG**
